# Dapagliflozin/Hesperidin Combination Mitigates Lipopolysaccharide-Induced Alzheimer’s Disease in Rats

**DOI:** 10.3390/ph16101370

**Published:** 2023-09-27

**Authors:** Maaly A. Abd Elmaaboud, Remon S. Estfanous, Aliaa Atef, Ahmed M. Kabel, Khalid A. Alnemari, Tamer M. Naguib, Shuruq E. Alsufyani, Hany W. Darwish, Hany H. Arab

**Affiliations:** 1 Department of Pharmacology, Faculty of Medicine, Tanta University, Tanta 31527, Egypt; maali_abdelmaboud@med.tanta.edu.eg; 2 Anatomy and Embryology Department, Faculty of Medicine, Tanta University, Tanta 31527, Egypt; remon.astfanous@med.tanta.edu.eg; 3 Department of Pathology, Faculty of Medicine, Tanta University, Tanta 31527, Egypt; aliaa.shamseldeen@med.tanta.edu.eg; 4 Taif Medical Center, Taif 26526, Saudi Arabia; khalid7alnemari@gmail.com; 5 Anesthesia and ICU Department, Faculty of Medicine, Tanta University, Tanta 31527, Egypt; tnaguib1eg@yahoo.com; 6 Department of Pharmacology and Toxicology, College of Pharmacy, Taif University, P.O. Box 11099, Taif 21944, Saudi Arabia; s.alsofyani@tu.edu.sa (S.E.A.); or hany.arab@pharma.cu.edu.eg (H.H.A.); 7 Department of Pharmaceutical Chemistry, College of Pharmacy, King Saud University, Riyadh 11451, Saudi Arabia; hdarwish@ksu.edu.sa; 8 Department of Biochemistry, Faculty of Pharmacy, Cairo University, Cairo 11562, Egypt

**Keywords:** dapagliflozin, hesperidin, lipopolysaccharide, Alzheimer’s disease, oxidative stress, inflammatory cascade, apoptosis, autophagy, rats

## Abstract

Alzheimer’s disease (AD) is the most common form of neurodegenerative disorders worldwide. Its pathologic features include massive neuroinflammation with abnormal deposition of β-amyloid peptide in the cerebral tissues leading to degeneration of the brain neurons. Adverse effects associated with the traditional drugs used for the treatment of this pathological condition have directed the research efforts towards searching for alternative effective agents with minimal adverse effects. The aim of this study was to elucidate the potential ameliorative effects of dapagliflozin and/or hesperidin on Alzheimer’s disease (AD) induced by lipopolysaccharide (LPS) injection in rats. In a rodent model of AD, the effect of dapagliflozin with or without hesperidin on the biochemical parameters and the behavioral tests as well as the histopathological parameters was determined. Each of dapagliflozin and hesperidin restored the behavioral tests to the reference values, augmented the antioxidant defense mechanisms, ameliorated the neuronal inflammatory responses, combatted the changes in Toll-like receptor-4 (TLR-4)/High-mobility group box 1 (HMGB1) protein signaling and receptors of advanced glycation end products (RAGE) levels, and restored the balance between the apoptotic signals and autophagy in the hippocampal tissues. Additionally, both agents exhibited an outstanding ability to combat LPS-induced perturbations in the histopathological and electron microscopic image of the brain tissues. These favorable effects were significantly encountered in the group treated with dapagliflozin/hesperidin combination when compared versus animals treated with either dapagliflozin or hesperidin. In conclusion, inhibition of the hippocampal HMGB1/TLR4/RAGE signaling, the pro-inflammatory axis, and apoptosis alongside augmentation of the antioxidant defenses and autophagy can be regarded as beneficial effects by which dapagliflozin/hesperidin combination may combat LPS-triggered AD.

## 1. Introduction

Alzheimer’s disease (AD) is one of the most prevalent forms of neurodegenerative disorders worldwide [1]. Its pathognomonic characteristics include abnormal deposition of β-amyloid peptide and formation of senile plaques in the brain associated with massive synaptic loss and degeneration of the cortical and subcortical neurons [2]. These changes were negatively reflected in the memory and cognitive functions with affection of behavior, orientation, and motor coordination [3]. The exact etiology of AD is not yet fully explored [4]. Nevertheless, the creation of a massive inflammatory state in the brain microenvironment was regarded as the principal triggering event for the pathologic changes of AD, as it perturbates the antioxidant cascade and elicits significant disturbances of autophagy/apoptosis balance [5].

High-mobility group box 1 (HMGB1) is a nuclear protein that has diverse activities in different biological systems [6]. Activation of Nod-like receptor protein 3 (NLRP3) inflammasome, which plays a crucial role in the inflammatory cascade, was reported to be the triggering factor for the production and release of HMGB1 from the macrophages [7]. This release is responsible for NLRP3 inflammasome-mediated cognitive impairment in a wide variety of neurological illnesses [8]. Recent reports stated that HMGB1 may play a fundamental role in the initiation and progression of neurodegenerative disorders, including AD [9]. HMGB1 was reported to bind directly to the receptors of advanced glycation end products (RAGE) and Toll-like receptor 4 (TLR4) [10]. This binding was found to be responsible for the initiation of the neuroinflammatory responses that eventually lead to the pathognomonic features of AD [11]. Additionally, recent studies postulated that the therapeutic regimens of AD that take HMGB1 as a potential target may be effective in halting AD progression [10,12].

The role of autophagy/apoptosis balance in the pathologic evidence of AD was thoroughly studied in recent research trials [13]. It was reported that cases with AD have significantly declined levels of autophagy markers relative to normal individuals [14]. Activation of the PI3KI/Akt/mTOR pathway and increased TLR4 expression in the neuronal tissues were reported to be responsible for the defective autophagy in AD [15]. In addition, TLR4 was proven to enhance the expression of the proapoptotic molecules with subsequent massive neurodegeneration [12].

Ki-67 is a nuclear antigen that is overexpressed in cases associated with active cell proliferation, hence, being considered a reliable marker of the response to systemic therapy in a wide range of systemic diseases [16]. Recent research has proven a strong relationship between Ki-67 expression and the pathogenesis of AD [17]. The cell cycle abnormalities that occur in AD were reported to be associated with overexpression of Ki-67 in the mature neurons which was proven to be directly linked to neuronal cell death [18].

As neuroinflammation is the key pathological event in neurodegenerative disorders including AD, various stimuli, including lipopolysaccharide (LPS), were utilized as a model of the inflammatory processes that play a key role in neurodegeneration [5]. LPS was reported to bind to certain receptors in the microglia and the astrocytes leading to the modulation of a number of intracellular molecules that are considered keystones in the inflammatory cascade in the neuronal tissues with the end result of massive neurodegeneration [19]. However, the neuropathological responses to LPS injection may be widely variable according to the route of administration, the dose, the frequency of injection, and the serotype of LPS [20].

Dapagliflozin is a sodium–glucose cotransporter 2 (SGLT2) inhibitor that ameliorates hyperglycemia in type 2 diabetes mellitus [21]. Recent reports suggested that dapagliflozin may exert potent neuroprotective effects in different animal models of neurological illnesses apart from its antidiabetic properties [22]. This might originate from its ability to combat the deleterious consequences of reactive oxygen species (ROS) released as a response to neurotoxic stimuli [23]. In addition, the modulatory effects of dapagliflozin on the different signaling pathways involved in the inflammatory cascade and apoptosis might impart a possible role for dapagliflozin in the mitigation of AD [24].

Hesperidin is a flavonoid that is found in considerable amounts in citrus fruits [25]. As it possesses a wide range of biological effects, the neuroprotective effects of hesperidin were investigated on a wide scale in scientific research [26]. Improvement of the neural growth factor levels and restoration of the antioxidant defenses were proven to be responsible for the beneficial neurological effects of hesperidin [27]. This together with its ability to combat the neuro-inflammatory stimuli and to inhibit the apoptotic pathways in the brain may make hesperidin a promising agent for management of the neurodegenerative disorders [28]. The current work was performed to elucidate the effects of the administration of dapagliflozin and/or hesperidin on AD induced by lipopolysaccharide (LPS) injection in rats and to explore the potential mechanisms that might underlie these effects.

## 2. Results

### 2.1. Dapagliflozin with or without Hesperidin Ameliorated the Changes in the Behavioral Tests Elicited by LPS Injection

**Figure 1:**
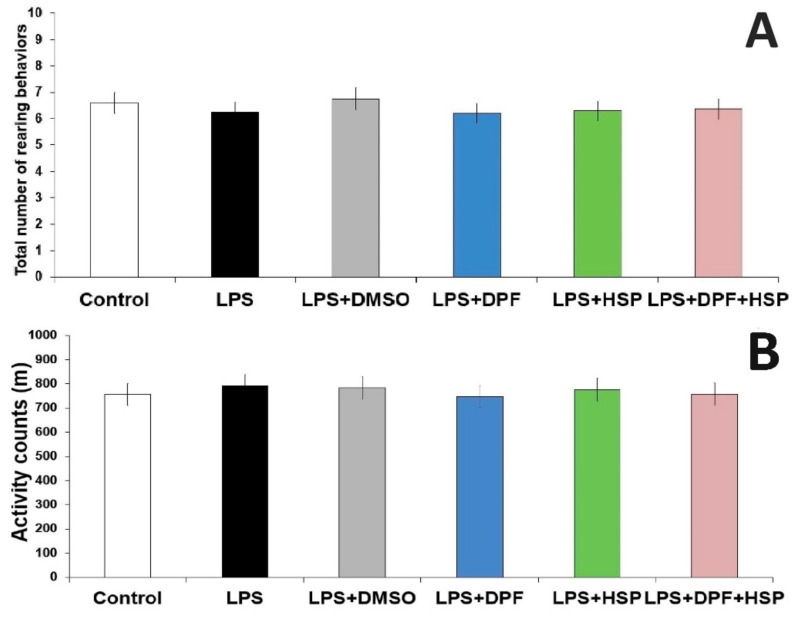
Effect of dapagliflozin and/or hesperidin on (**A**) the total number of rearing behaviors and (**B**) the locomotor activity in rats injected with lipopolysaccharide (LPS) (Mean ± SD); LPS (lipopolysaccharide), DMSO (dimethyl sulfoxide), DPF (dapagliflozin), and HSP (hesperidin).

In open field locomotion test, the locomotor activity and the total number of rearing behaviors were not significantly affected by the administration of any of the used drugs relative to the control group (Figure 1).

**Figure 2:**
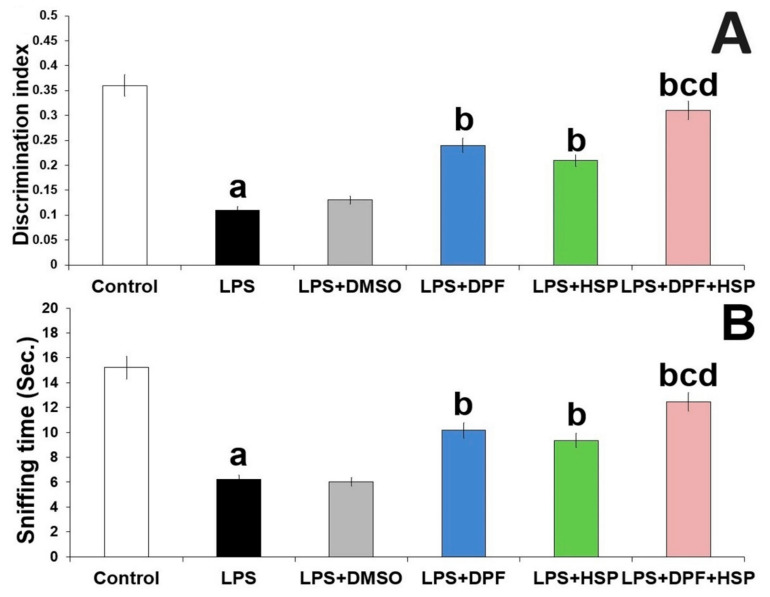
Effect of dapagliflozin and/or hesperidin on (**A**) the discrimination index and (**B**) the sniffing time in rats injected with lipopolysaccharide (Mean ± SD). ^a^ *p*-value < 0.05 versus the control group; ^b^ *p*-value < 0.05 versus LPS-injected group; ^c^ *p*-value < 0.05 versus LPS rats treated with dapagliflozin; ^d^ *p*-value < 0.05 versus LPS rats treated with hesperidin. LPS (lipopolysaccharide), DMSO (dimethyl sulfoxide), DPF (dapagliflozin), and HSP (hesperidin).

During the testing phase of the object recognition task, rats injected with LPS exhibited significant shortening in the sniffing time (60.78% decrease; 95% confidence interval (CI): from 7.9 to 10.1) and significant decline in the discrimination index (66.67% decrease; 95% CI: from 0.22 to 0.28) when compared versus the control group. Both the sniffing time and the discrimination index were not significantly influenced by the administration of DMSO to LPS-injected rats when compared versus the group that received LPS alone. The effects of LPS injection on the object recognition task were significantly abrogated with administration of either dapagliflozin (98% increase; 95% CI: from −0.15 to −0.11 for the discrimination index and 78% increase; 95% CI: from −5.05 to −2.85 for the sniffing time) or hesperidin (79% increase; 95% CI: from −0.12 to −0.08 for the discrimination index and 66% increase; 95% CI: from −4.25 to −2.05 for the sniffing time). Interestingly, the most beneficial effects were obviously detected in the group treated with the dapagliflozin/hesperidin combination when compared versus animals treated with each of these agents alone (Figure 2).

**Figure 3:**
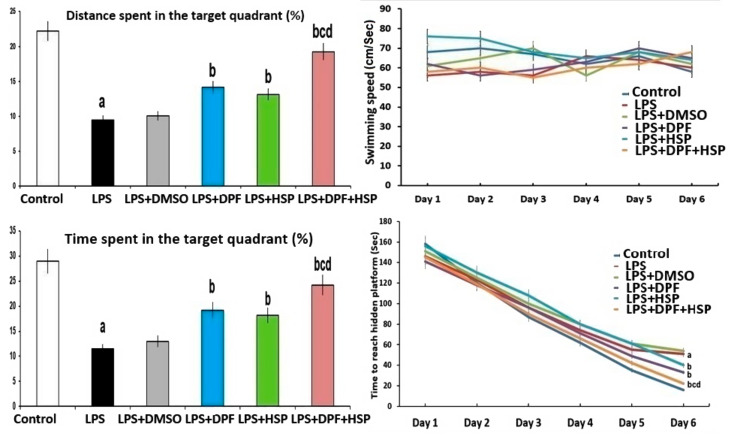
Effect of dapagliflozin and/or hesperidin on Morris Water Maze test in rats injected with lipopolysaccharide (Mean ± SD). ^a^ *p*-value < 0.05 versus the control group; ^b^ *p*-value < 0.05 versus LPS-injected group; ^c^ *p*-value < 0.05 versus LPS rats treated with dapagliflozin; ^d^ *p*-value < 0.05 versus LPS rats treated with hesperidin. LPS (lipopolysaccharide), DMSO (dimethyl sulfoxide), DPF (dapagliflozin), and HSP (hesperidin).

In the Morris Water Maze test, the mean swimming speed was not significantly affected by the administration of the different agents relative to the control group. Meanwhile, the time taken to reach a hidden platform was significantly prolonged in the group treated with LPS alone relative to the control group (2.23-fold increase; 95% CI: from −43.34 to −32.26). Nevertheless, a significant decrease in the time and the distance spent in the target quadrant was obviously observed in LPS-injected animals when compared versus the control group (62.7% decrease; 95% CI: from 6.44 to 27.42 for the time and 60.9% decrease; 95% CI: from 11.55 to 14.67 for the distance spent in the target quadrant). DMSO administered to LPS-injected rats did not significantly influence the results of the Morris Water Maze test when compared to animals treated with LPS alone. Interestingly, treatment with either dapagliflozin or hesperidin significantly diminished the time taken to reach a hidden platform (38.71% decrease; 95% CI: from 13.81 to 25.55 for dapagliflozin and 29.82% decrease; 95% CI: from 7.66 to 19.9 for hesperidin), significantly prolonged the time spent in the target quadrant (58.33% increase; 95% CI: from −17.42 to 3.5 for dapagliflozin and 49.25% increase; 95% CI: from −15.49 to 5.43 for hesperidin), and significantly increased the distance relative to the animal group treated with LPS alone (65.56% increase; 95% CI: from −6.72 to −3.6 for dapagliflozin and 55.78% increase; 95% CI: from −5.52 to −2.39 for hesperidin) but dapagliflozin/hesperidin combination group exhibited the most favorable outcomes (Figure 3).

### 2.2. Dapagliflozin with or without Hesperidin Mitigated the Effect of LPS Administration on the Antioxidant Status and Nuclear Factor Erythroid 2-Related Factor 2 (Nrf2) Content of the Hippocampal Tissues

**Figure 4:**
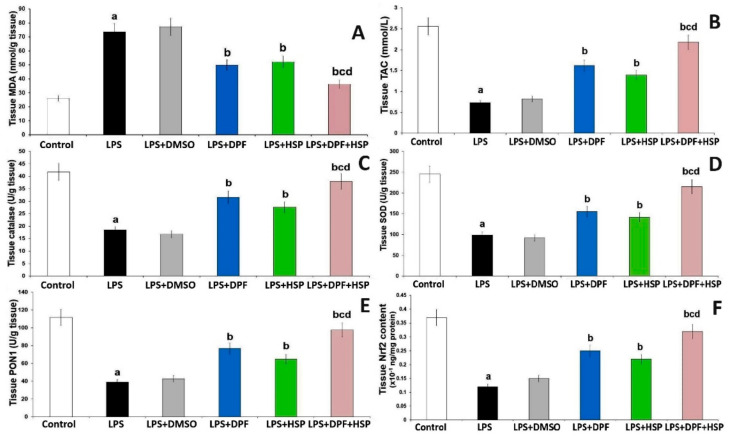
Effect of dapagliflozin and/or hesperidin on (**A**) malondialdehyde, (**B**) total antioxidant capacity, (**C**) catalase, (**D**) superoxide dismutase, (**E**) paraoxonase-1, and (**F**) Nrf2 content of the hippocampal tissues of rats injected with lipopolysaccharide (LPS) (Mean ± SD). ^a^ *p*-value < 0.05 versus the control group; ^b^ *p*-value < 0.05 versus LPS-injected group; ^c^ *p*-value < 0.05 versus LPS rats treated with dapagliflozin; ^d^ *p*-value < 0.05 versus LPS rats treated with hesperidin. LPS (lipopolysaccharide), DMSO (dimethyl sulfoxide), DPF (dapagliflozin), and HSP (hesperidin).

Injection of LPS created a state of oxidative damage to the hippocampal tissues manifested by significant elevation of malondialdehyde (MDA) levels (1.88-fold increase; 95% CI: from −63.62 to −31.38) with significant decrement in tissue antioxidant enzymes (57.15% decrease; 95% CI: from 14.51 to 32.27 for catalase, 61.02% decrease; 95% CI: from 95.92 to 197.5 for superoxide dismutase, and 67.11% decrease; 95% CI: from 45.02 to 93.66 for paraoxonase-1), the total antioxidant capacity (73.17% decrease; 95% CI: from 1.38 to 2.28), and Nrf2 content (67.57% decrease; 95% CI: from 0.18 to 0.32) relative to the control group. DMSO administered to LPS-injected rats did not induce any significant changes in the antioxidant status and Nrf2 content of the hippocampal tissues when compared to rats treated with LPS alone. Animals treated with either dapagliflozin or hesperidin exhibited significant improvement in the levels of the antioxidant enzymes with significant increase in the total antioxidant capacity (1.22-fold increase; 95% CI: from −1.34 to −0.44 for dapagliflozin and 1.08 folds increase; 95% CI: from −1.11 to −0.21 for hesperidin) and Nrf2 content (98.23% increase; 95% CI: from −0.2 to −0.06 for dapagliflozin and 83.41% increase; 95% CI: from −0.17 to −0.03 for hesperidin) and significant decline in MDA level (33.54% decrease; 95% CI: from 7.69 to 39.9 for dapagliflozin and 29.97% decrease; 95% CI: from 5.32 to 37.56 for hesperidin) in the hippocampal tissues relative to animals that received LPS alone. These changes were maximally remarkable in the group treated with the dapagliflozin/hesperidin combination when compared with the groups treated with each of these agents alone (Figure 4).

### 2.3. Dapagliflozin with or without Hesperidin Mitigated the Effect of LPS Injection on the Inflammatory Consequences in the Hippocampal Tissues

**Figure 5:**
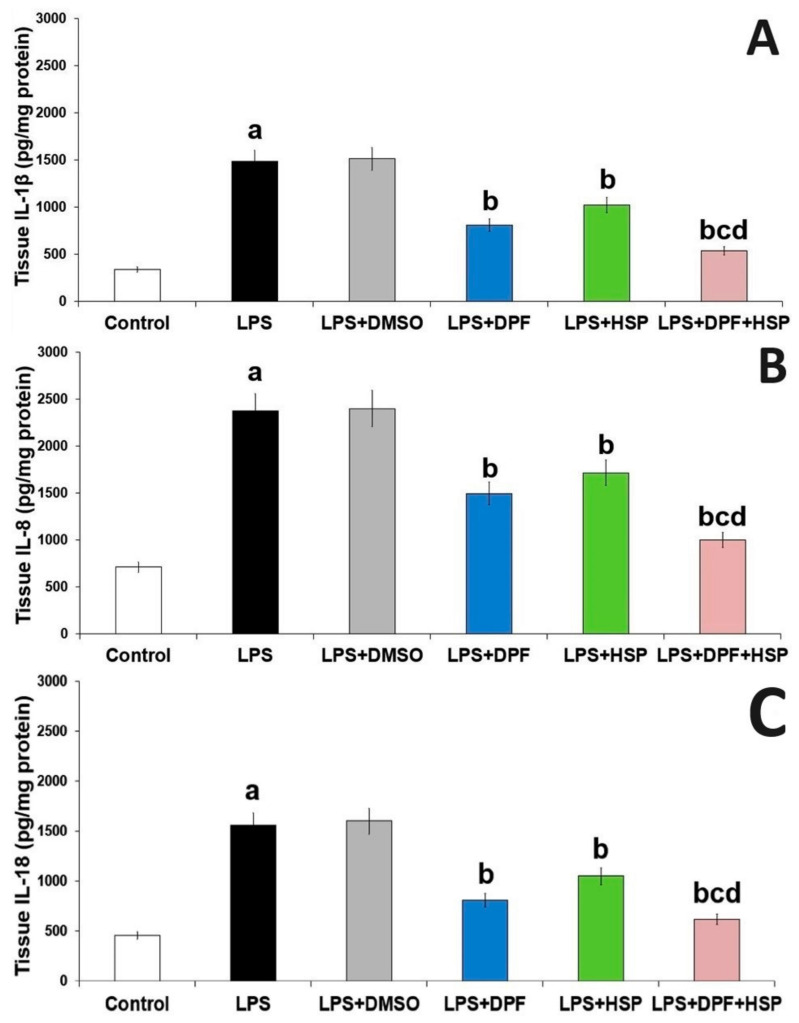
Effect of dapagliflozin and/or hesperidin on (**A**) IL-1β, (**B**) IL8, and (**C**) IL-18 levels in the hippocampal tissues of rats injected with lipopolysaccharide (Mean ± SD). ^a^ *p*-value < 0.05 versus the control group; ^b^ *p*-value < 0.05 versus LPS-injected group; ^c^ *p*-value < 0.05 versus LPS rats treated with dapagliflozin; ^d^ *p*-value < 0.05 versus LPS rats treated with hesperidin. LPS (lipopolysaccharide), DMSO (dimethyl sulfoxide), DPF (dapagliflozin), and HSP (hesperidin).

**Figure 6:**
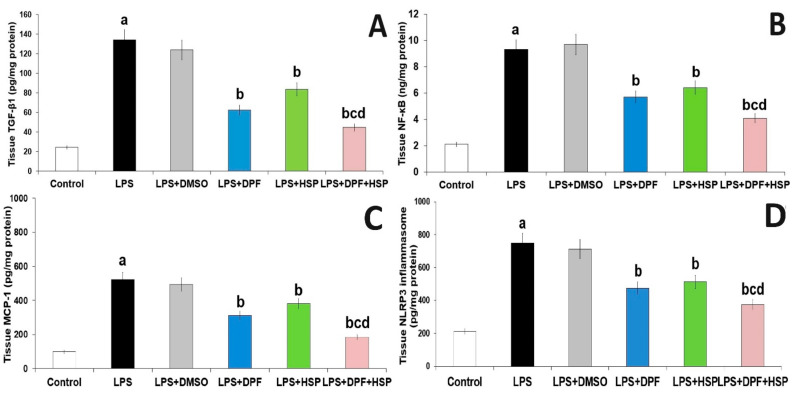
Effect of dapagliflozin and/or hesperidin on (**A**) TGF-β1, (**B**) NF-κB, (**C**) MCP-1, and (**D**) NLRP3 inflammasome in the hippocampal tissues of rats injected with lipopolysaccharide (Mean ± SD). ^a^ *p*-value < 0.05 versus the control group; ^b^ *p*-value < 0.05 versus LPS-injected group; ^c^ *p*-value < 0.05 versus LPS rats treated with dapagliflozin; ^d^ *p*-value < 0.05 versus LPS rats treated with hesperidin. LPS (lipopolysaccharide), DMSO (dimethyl sulfoxide), DPF (dapagliflozin), and HSP (hesperidin).

Injection of LPS in the current work augments inflammation in the brain microenvironment manifested by significantly elevated tissue levels of transforming growth factor beta 1 (TGF-β1) (4.58-fold increase; 95% CI: from −141.7 to −78.17), NLRP3 inflammasome (2.55-fold increase; 95% CI: from −687.8 to −387.2), and nuclear factor kappa B (NF-κB) (3.39-fold increase; 95% CI: from −9.54 to −4.86) with subsequent significant increase in interleukin (IL)-1β (3.34-fold increase; 95% CI: from −1472 to −814.1), IL8 (2.33-fold increase; 95% CI: from −2099 to −1225), IL-18 (2.43-fold increase; 95% CI: from −1463 to −753.6), and monocyte chemoattractant protein-1 (MCP-1) (4.33-fold increase; 95% CI: from −563.9 to −285.2) in the hippocampal tissues when compared to the control group. DMSO administered to LPS-injected rats did not significantly affect the levels of the aforementioned inflammatory mediators relative to rats treated with LPS alone. Interestingly, the administration of either dapagliflozin or hesperidin in the current study reversed these changes in the aforementioned biochemical parameters while the administration of the dapagliflozin/hesperidin combination restored the tissue levels of the aforementioned inflammatory mediators to approximate the reference values (Figure 5 and Figure 6).

### 2.4. Dapagliflozin with or without Hesperidin Abrogated the Effect of LPS Injection on TLR4/RAGE/HMGB1 Signaling in the Hippocampal Tissues

**Figure 7:**
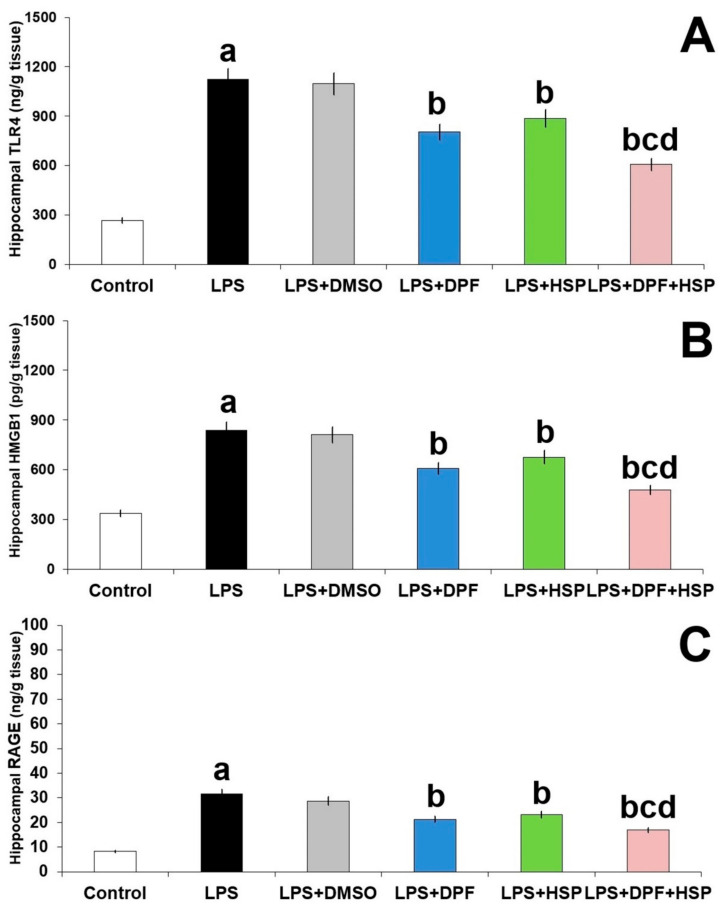
Effect of dapagliflozin and/or hesperidin on (**A**) TLR4, (**B**) HMGB1, and (**C**) RAGE in the hippocampal tissues of rats injected with lipopolysaccharide (Mean ± SD). ^a^ *p*-value < 0.05 versus the control group; ^b^ *p*-value < 0.05 versus LPS-injected group; ^c^ *p*-value < 0.05 versus LPS rats treated with dapagliflozin; ^d^ *p*-value < 0.05 versus LPS rats treated with hesperidin. LPS (lipopolysaccharide), DMSO (dimethyl sulfoxide), DPF (dapagliflozin), HSP (hesperidin).

Significant augmentation of TLR4/RAGE/HMGB1 signaling in the hippocampal tissues was noticed in the group treated with LPS alone relative to the control group (3.31-fold increase; 95% CI: from −966.9 to −745.6 for TLR4, 2.76-fold increase; 95% CI: from −26.4 to −20.02 for RAGE, and 1.61-fold increase; 95% CI: from −575.5 to −431.2 for HMGB1). DMSO administered to LPS-injected animals did not significantly affect the TLR4/RAGE/HMGB1 axis in the hippocampal tissues relative to animals injected with LPS alone. Administration of either dapagliflozin or hesperidin in the current study had the ability to combat the changes induced by LPS injection in the tissue levels of TLR4 (31.35% decrease; 95% CI: from 197.3 to 441.4 for dapagliflozin and 23.21% decrease; 95% CI: from 112.7 to 361.1 for hesperidin); RAGE (28.56% decrease; 95% CI: from 6.78 to 13.62 for dapagliflozin and 25.23% decrease; 95% CI: from 4.88 to 11.74 for hesperidin); and HMGB1 (26.83% decrease; 95% CI: from 158.9 to 303.1 for dapagliflozin and 20.73% decrease; 95% CI: from 91.19 to 235.5 for hesperidin) but animals treated with the dapagliflozin/hesperidin combination had the most successful results (Figure 7).

### 2.5. Dapagliflozin with or without Hesperidin Ameliorated the Effect of LPS Injection on PI3K/Akt/mTOR Axis in the Hippocampal Tissues

**Figure 8:**
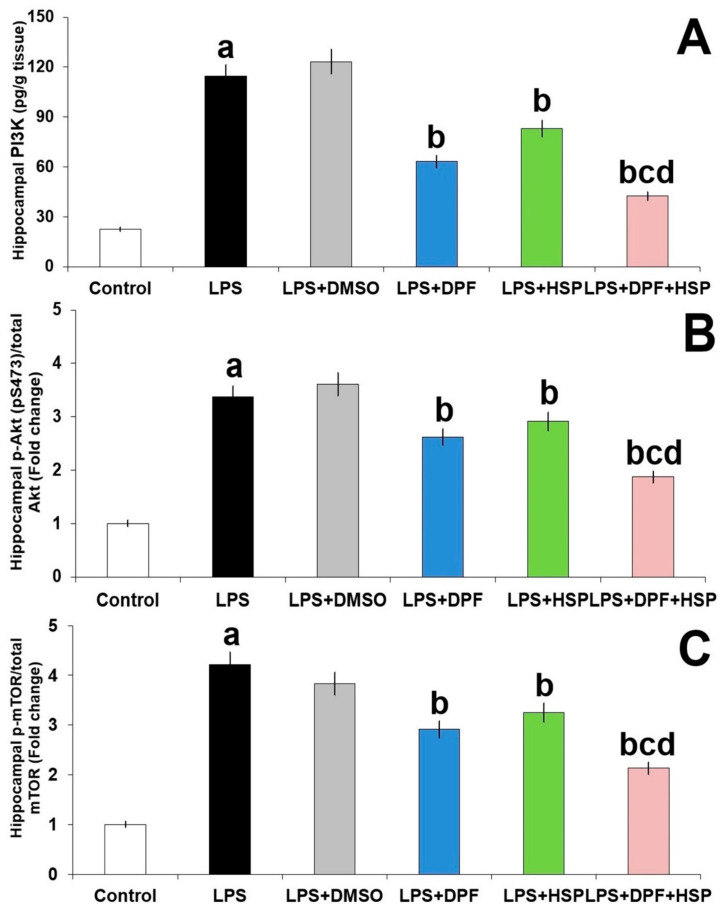
Effect of dapagliflozin and/or hesperidin on (**A**) PI3K, (**B**) p-Akt/total Akt, and (**C**) p-mTOR/total m-TOR levels in the hippocampal tissues of rats injected with lipopolysaccharide (Mean ± SD). ^a^ *p*-value < 0.05 versus the control group; ^b^ *p*-value < 0.05 versus LPS-injected group; ^c^ *p*-value < 0.05 versus LPS rats treated with dapagliflozin; ^d^ *p*-value < 0.05 versus LPS rats treated with hesperidin. LPS (lipopolysaccharide), DMSO (dimethyl sulfoxide), DPF (dapagliflozin), and HSP (hesperidin).

As depicted in Figure 8, LPS augmented PI3K/Akt/mTOR signaling in the hippocampal tissues, indicated by a significant elevation in PI3K levels (4.14-fold increase; 95% CI: from −102.7 to −81.52), a significant increase in Akt phosphorylation to the total Akt protein (2.41-fold increase; 95% CI: from −2.67 to −2.08), and significant augmentation of mTOR phosphorylation to the total mTOR protein (3.15-fold increase; 95% CI: from −3.57 to −2.88) relative to the control animals. Administration of DMSO did not significantly affect LPS-enhanced perturbations in PI3K/Akt/mTOR signaling in the hippocampal tissues. However, each of dapagliflozin and hesperidin exhibited an amazing ability to mitigate the effects of LPS injection on the PI3K/Akt/mTOR axis in the hippocampal tissues with the most beneficial effects in favor of dapagliflozin/hesperidin combination.

### 2.6. Dapagliflozin with or without Hesperidin Reversed the Perturbations in Autophagy Induced by LPS Injection in the Hippocampal Tissues

**Figure 9:**
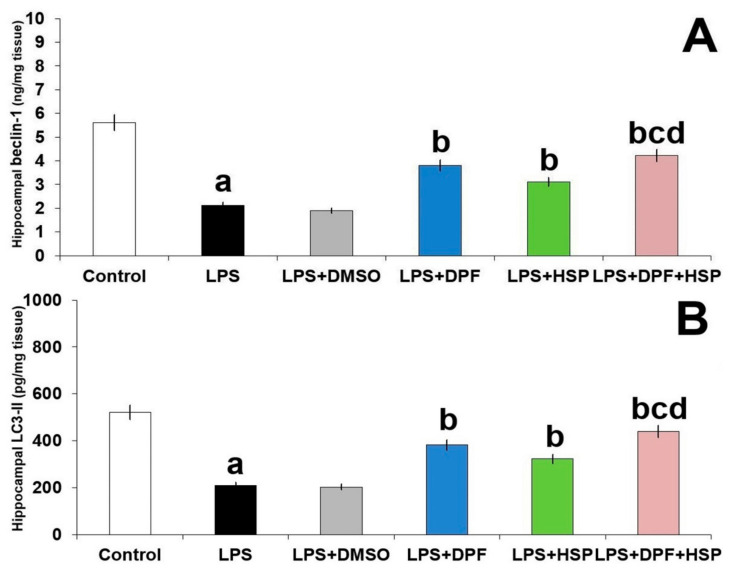
Effect of dapagliflozin and/or hesperidin on (**A**) beclin-1 and (**B**) LC3-II in the hippocampal tissues of rats injected with lipopolysaccharide (Mean ± SD). ^a^ *p*-value < 0.05 versus the control group; ^b^ *p*-value < 0.05 versus LPS-injected group; ^c^ *p*-value < 0.05 versus LPS rats treated with dapagliflozin; ^d^ *p*-value < 0.05 versus LPS rats treated with hesperidin. LPS (lipopolysaccharide), DMSO (dimethyl sulfoxide), DPF (dapagliflozin), and HSP (hesperidin).

The antagonistic effects of LPS to autophagy in the hippocampal tissues were revealed in the present study by significant diminution of the tissue levels of LC3-II and beclin-1 (62.11% decrease; 95% CI: from 273.2 to 349.8 and 59.92% decrease; 95% CI: from 3.01 to 3.95, respectively) relative to the control group. Administration of DMSO did not significantly affect the autophagy markers relative to animals treated with LPS alone. A significant increase in the levels of the aforementioned autophagy markers was noticed in the groups treated with either dapagliflozin (90.69% increase; 95% CI: from −209.7 to −133.1 for LC3-II and 91.24% increase; 95% CI: from −2.07 to −1.31 for beclin-1) or hesperidin (73.29% increase; 95% CI: from −150.4 to −73.77 for LC3-II and 65.21% increase; 95% CI: from −1.299 to −0.7 for beclin-1), but the maximal response was achieved in the group treated with dapagliflozin/hesperidin combination (Figure 9).

### 2.7. Dapagliflozin with or without Hesperidin Combatted the Apoptotic Changes Elicited by Lipopolysaccharide Injection in the Hippocampal Tissues

**Figure 10:**
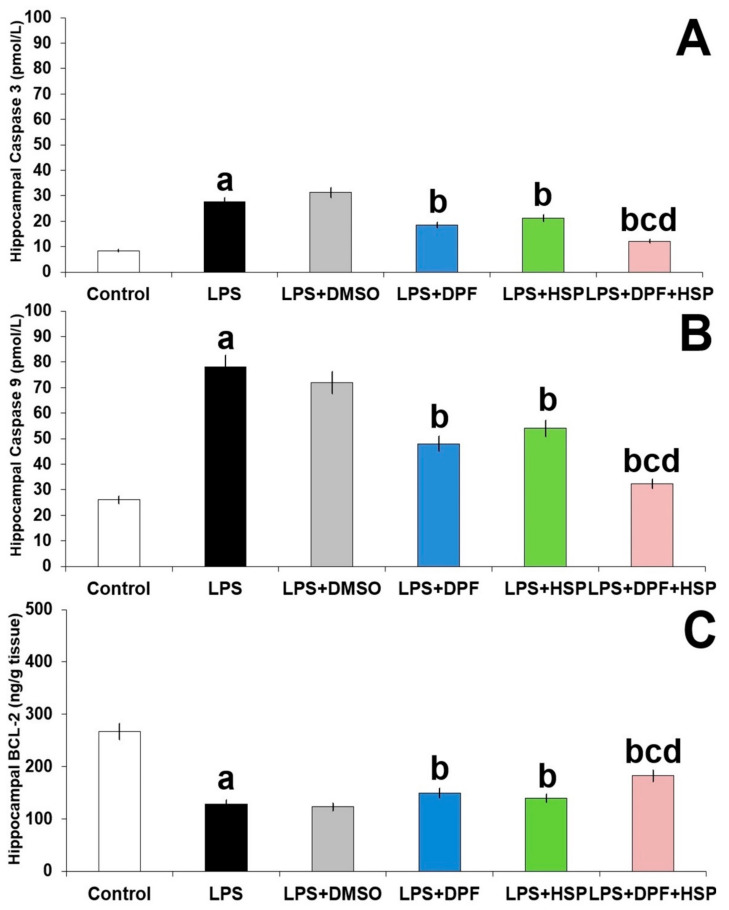
Effect of dapagliflozin and/or hesperidin on (**A**) caspase 3, (**B**) caspase 9, and (**C**) BCL-2 in the hippocampal tissues of rats injected with lipopolysaccharide (Mean ± SD). ^a^ *p*-value < 0.05 versus the control group; ^b^ *p*-value < 0.05 versus LPS-injected group; ^c^ *p*-value < 0.05 versus LPS rats treated with dapagliflozin; ^d^ *p*-value < 0.05 versus LPS rats treated with hesperidin. LPS (lipopolysaccharide), DMSO (dimethyl sulfoxide), DPF (dapagliflozin), and HSP (hesperidin).

In the current work, apoptosis was induced by injection of LPS, manifested as significant elevation in caspase 3 and caspase 9 levels (2.18-fold increase; 95% CI: from −21.41 to −16.99 and 1.88-fold increase; 95% CI: from −59.91 to −44.69, respectively) with significant diminution in BCL-2 expression (48.11% decrease; 95% CI: from 82.25 to 114.7) in the hippocampal tissues relative to the control group. Apoptosis in the hippocampal tissues of LPS-injected animals was not affected by the administration of DMSO. Nevertheless, the levels of caspase 3 and caspase 9 were dramatically diminished and the expression of the antiapoptotic protein BCL-2 was significantly improved with administration of either dapagliflozin (25.93% decrease; 95% CI: from 6.99 to 11.41 for caspase 3, 35.89% decrease; 95% CI: from 22.08 to 38.32 for caspase 9, and 24.15% increase; 95% CI: from −34.35 to −1.85 for BCL-2) or hesperidin (17.84% decrease; 95% CI: from 4.19 to 8.61 for caspase 3, 26.92% decrease; 95% CI: from 15.81 to 32.39 for caspase 9, and 18.53% increase; 95% CI: from −27.15 to 5.35 for BCL-2), but the group treated with their combination expressed the most favorable results (Figure 10).

### 2.8. Dapagliflozin with or without Hesperidin Reversed the Changes in the Histopathological Morphology of the Hippocampus and the Frontal Lobe Induced by LPS Injection

**Figure 11:**
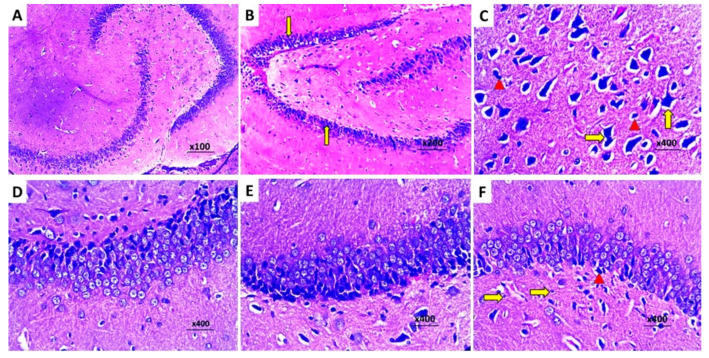
Sections from the hippocampus stained with hematoxylin and eosin of (**A**) the control rats showing multiple tightly packed layers of pyramidal cells of different sizes with polygonal cell bodies, vesicular nuclei, and prominent nucleoli; (**B**) LPS-injected rats exhibiting significantly diminished pyramidal cell layer thickness with multiple dystrophic neurons showing apoptotic changes with shrunken hyperchromatic pyknotic nuclei and chromatin condensation (arrows); (**C**) LPS-injected rats treated with DMSO exhibiting large-sized dispersed multipolar neurons (arrows) with neurons of smaller size in between (arrow heads); (**D**) LPS-injected rats treated with dapagliflozin exhibiting moderate decline in the number of the nuclei that showed apoptotic changes with significantly increased number of the normal nuclei; (**E**) LPS-injected rats treated with hesperidin showing significantly decreased number of the apoptotic neurocytic nuclei with increased number of the normal nuclei; (**F**) LPS-injected rats treated with dapagliflozin/hesperidin combination exhibiting marked increase in the number of the normal neurons (arrows) with scanty apoptotic neurons in between (arrow head).

**Figure 12:**
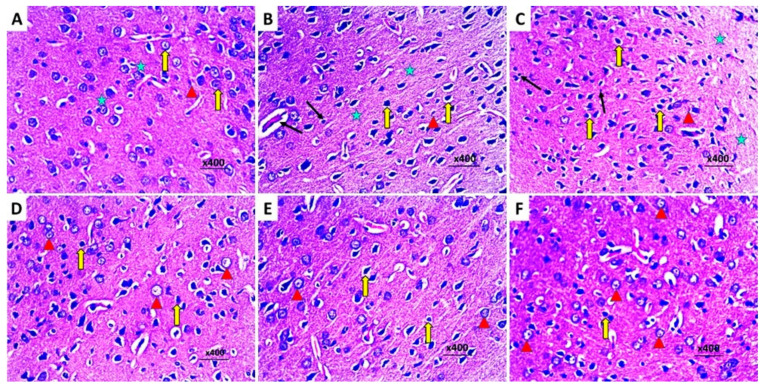
Sections from the frontal lobe stained with hematoxylin and eosin of (**A**) the control group exhibiting normal neurons with polygonal cell bodies, large vesicular nuclei, prominent nucleoli and normal distribution of Nissl’s granules (arrow), microglia (arrow head), and scattered macrophages (asterix); (**B**) LPS-injected rats exhibiting significant neurocytic dystrophy with shrunken hyperchromatic pyknotic nuclei with abundant chromatin condensation (thick arrows), massive infiltration with lymphocytes (thin arrows), vascular congestion (arrow head), and spongiform necrosis (asterix); (**C**) LPS-injected rats treated with DMSO showing significant dystrophy of the neurons, shrunken hyperchromatic nuclei (thick arrows), venous stasis (arrow head), lymphocytic infiltration (thin arrows), and scattered areas of spongiform necrosis (asterix); (**D**) LPS-injected rats treated with dapagliflozin exhibiting decreased number of the apoptotic neurons (arrows), increased near normal neurons with central large vesicular nuclei, and peripheral distribution of Nissl’s granules (arrow heads); (**E**) LPS-injected rats treated with hesperidin showing scanty apoptotic neurons (arrows), abundance of the apparently normal neurocytes, with peripherally placed Nissl’s granules (arrow heads); (**F**) LPS-injected rats treated with dapagliflozin/hesperidin combination showing marked decrease in the number of the neurons with dystrophic changes with shrunken hyperchromatic nuclei (arrow) and increased near normal neurons with central large vesicular nuclei, containing one or more nucleoli, and peripheral distribution of Nissl’s granules (arrow heads).

The control group exhibited a normal histological picture of the brain tissues with normal hippocampus (Figure 11A) and cerebral cortex (Figure 12A). Injection of LPS induced obvious neurodegeneration and disorganization of the brain tissues, and the activation of microglia, with a significant decline in the number of viable neurons relative to the control group (Figure 11B and Figure 12B). Administration of DMSO did not significantly affect the histopathological findings created by LPS administration (Figure 11C and Figure 12C). In contrast, administration of either dapagliflozin or hesperidin elicited significant improvement of the histopathological picture with a noticeable increase in the number of normal polygonal neurons and a significant decline in the number of dystrophic apoptotic neurons (Figure 11D,E and Figure 12D,E), but the use of the dapagliflozin/hesperidin combination exhibited the most promising results (Figure 11F and Figure 12F).

### 2.9. Dapagliflozin with or without Hesperidin Downregulated the Expression of Ki-67 in the Hippocampus and the Frontal Lobe Induced by LPS Injection

**Figure 13:**
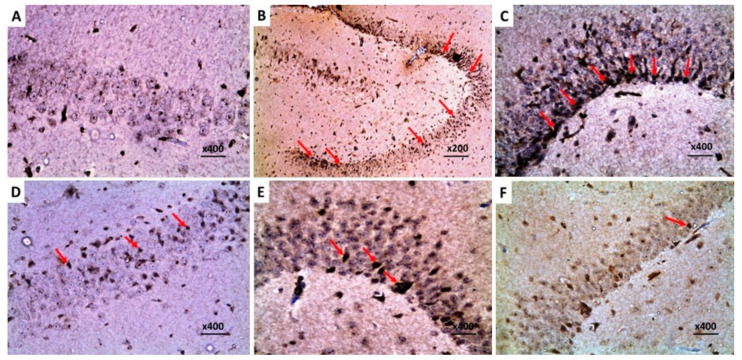
A photomicrograph of immunohistochemical staining of Ki-67 in the hippocampus of (**A**) the control rats exhibiting negative immunoreactivity of the pyramidal cells to Ki-67; (**B**,**C**) LPS-injected rats and LPS-injected rats treated with DMSO respectively showing strong positive nuclear immunoreactivity to Ki-67 (arrows); (**D**,**E**) LPS-injected rats treated with dapagliflozin and hesperidin, respectively, revealing moderate positive nuclear immunoreactivity to Ki-67 (arrows); (**F**) LPS-injected rats treated with dapagliflozin/hesperidin combination revealing weak positive immunoexpression of Ki-67 in the neurocytic nuclei (arrow) (Streptavidin biotin ×400).

**Figure 14:**
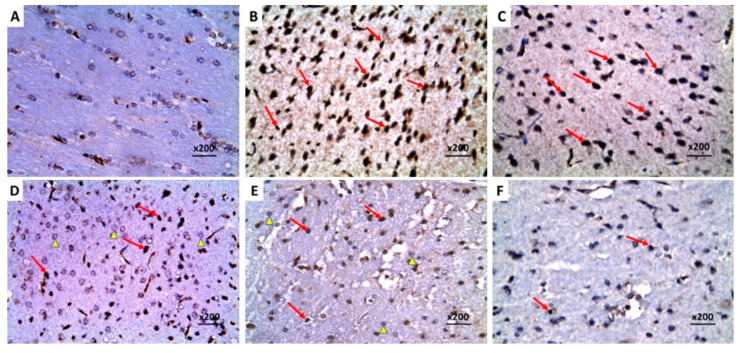
A photomicrograph of immunohistochemical staining of Ki-67 in the frontal lobe of (**A**) the control rats showing negative immunoexpression of Ki-67 in the neurocytic nuclei; (**B**,**C**) LPS-injected rats and LPS-injected rats treated with DMSO, respectively, exhibiting strong positive nuclear immunoreactivity to Ki-67 (arrows); (**D**,**E**) LPS-injected rats treated with dapagliflozin and hesperidin respectively showing moderate nuclear immunostaining of Ki-67 (arrows); (**F**) LPS-injected rats treated with dapagliflozin/hesperidin combination showing mild immunoreactivity of the neurocytic nuclei to Ki-67 (arrows) (Streptavidin biotin ×200).

The significant increase in the immunostaining of Ki-67 in the brain tissues of animals treated with LPS alone (Figure 13B and Figure 14B) was not significantly affected by DMSO administration (Figure 13C and Figure 14C), but it was significantly mitigated in the groups treated with either dapagliflozin or hesperidin (Figure 13D,E and Figure 14D,E). The most beneficial effects on Ki-67 immunoexpression were revealed in LPS-injected animals treated with a dapagliflozin/hesperidin combination relative to LPS animals treated with each of these agents alone (Figure 13F and Figure 14F).

### 2.10. Dapagliflozin with or without Hesperidin Mitigated the Electron Microscopic Morphologic Changes of the Frontal Lobe Elicited by LPS Injection

**Figure 15:**
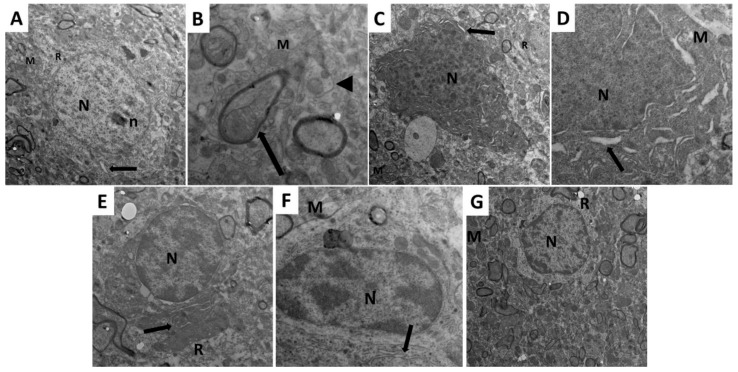
An electron micrograph of ultrathin sections in the cerebral cortex from (**A**) the control group revealing the pyramidal cell with large rounded regular nucleus (N) and prominent nucleolus (n). The cytoplasm shows normal mitochondria with normal cristae pattern (M), rough endoplasmic reticulum (arrow), and multiple free ribosomes (R) (TEM; ×1500 direct magnification); (**B**) the control group showing a myelinated axon with regular smooth contour of its myelin sheath (arrow) and an unmyelinated axon (arrow head). Normal-shaped mitochondria with normal cristae pattern (M) appear within their axoplasm (TEM; ×5000); (**C**) LPS-injected rats showing the pyramidal cell with markedly shrunken irregular nucleus with marked chromatin condensation (N). The cytoplasm shows swollen mitochondria with markedly destructed cristae (M), dilated rough endoplasmic reticulum (RER) (arrow) and scanty free ribosomes (R) (TEM; ×2000); (**D**) LPS-injected rats treated with DMSO exhibiting the pyramidal cell with markedly shrunken irregular nucleus, marked chromatin condensation (N) and the cytoplasm shows markedly dilated RER (arrow) and swollen mitochondria with disrupted cristae (M) (TEM; ×4000); (**E**) LPS-injected rats treated with dapagliflozin with the pyramidal cell nucleus regained its normal contour and chromatin distribution (N) with mildly dilated RER (Arrow) and scanty ribosomes (R) (TEM; ×2500); (**F**) LPS-injected rats treated with hesperidin in which the pyramidal cell nucleus regained its normal contour and chromatin distribution (N) with mildly dilated RER (arrow) and swollen mitochondria with mild disruption of the cristae (M) (TEM; ×4000 direct magnification); (**G**) LPS-injected rats treated with dapagliflozin/hesperidin combination showing the pyramidal cell with apical dendrites in which the nucleus regained its regular contour and chromatin distribution (N). The cytoplasm shows apparently normal mitochondria (M) and multiple free ribosomes (R) (TEM; ×1500).

In the frontal lobe of the brains of the control group, the pyramidal cells were prominent with their characteristic long apical dendrites, large rounded nuclei, and prominent nucleoli (Figure 15A). The cytoplasm of the pyramidal cells of the control group showed normally shaped mitochondria and rough endoplasmic reticulum (RER) (Figure 15B). The LPS-injected group showed significant dysmorphic features of the pyramidal cells with irregular shrunken nuclei, swollen mitochondria with destructed cristae, and markedly dilated RER (Figure 15C). These dysmorphic features induced by LPS injection were not significantly affected by the administration of DMSO (Figure 15D). Administration of dapagliflozin with or without hesperidin restored the normal features of the pyramidal cells with apparently normal nuclei, mitochondria, and RER (Figure 15E,F), with the appearance of the most favorable results in the group treated with dapagliflozin/hesperidin combination (Figure 15G).

## 3. Discussion

The role of Nrf2 signaling in the pathogenesis of neurodegenerative disorders has been thoroughly studied in the last decade [29,30]. Significant expression of the oxidative stress markers in the brain of patients with neurodegenerative disorders was accompanied by significant decrement in the tissue Nrf2 levels and was positively correlated with the severity of neurodegeneration [31]. This was associated with significant abrogation of the activity of the antioxidant enzymes with subsequent affection of the histopathological picture of the brain tissues [32]. This was evident in the current study where LPS induced a significant decrease in Nrf2 content associated with significant decrement of the antioxidant defenses in the brain when compared against the control rats. Meanwhile, significant elevation of tissue MDA was noted in rats injected with LPS alone when compared against the control rats. These results were ameliorated in the LPS group treated with dapagliflozin which was proven to activate the signaling pathways in which Nrf2 is involved, and hence potentiates the antioxidant defenses and combats the deleterious effects of reactive oxygen species (ROS) in the central nervous system [33]. Additionally, the favorable effects of hesperidin on the brain Nrf2 with subsequent increases in the tissue’s total antioxidant capacity were noticed in the current work. This was in agreement with Ikram et al. [34] where hesperidin demonstrated significant potentiating effects on Nrf2 expression and the antioxidant enzymes. Additionally, Kim et al. [35] stated that hesperidin may modulate the cellular mechanisms that keep the pro-oxidant/antioxidant balance in the biological systems, including CNS, with the end result of enhancing the activity of the antioxidant defenses.

Overexpression of TLR4 was considered the main trigger for the inflammatory changes encountered in AD via both direct and indirect mechanisms [36]. The direct mechanisms might be mediated via enhancement of NLRP3 inflammasome and its downstream mediators which subsequently induce diffuse neuroinflammation in the brain with increased generation of the proinflammatory cytokines with the net result of massive neurodegeneration [37]. The indirect mechanisms are mediated via increased expression of HMGB1 and RAGE in the brain which were reported to represent key mediators in the pathogenesis of AD [11]. Recent reports have proven that HMGB1 induces chemotaxis in AD via its interaction with chemokine CXCL12, which in turn enhances the production of IL-1β, IL-6, and TNF-α in the hippocampal neurons [38]. In addition, HMGB1 was proven to inhibit amyloid-β (Aβ) peptide clearance from the rat hippocampus by inhibiting microglial phagocytosis, thus potentiating Aβ-induced neurodegeneration in AD [11]. Moreover, the role of HMGB1 in AD was attributed to its ability to enhance the RAGE/NF-κB cascade, with subsequent massive neuroinflammation [39]. These mechanisms were evident in the current work where LPS significantly enhanced TLR4/RAGE/HMGB1 signaling which was associated with increased tissue levels of TGF-β1, NLRP3 inflammasome, NF-κB, and the proinflammatory cytokines relative to the control group.

The findings of the present study are in the same line with recent studies that supported the hypothesis that the neuroprotective effects of dapagliflozin are due to the modulation of TLR4/RAGE/HMGB1 signaling with subsequent affection of the inflammatory cascade [40]. Feijóo-Bandín et al. [41] reported that the regulatory role of SGLT2 inhibitors, including dapagliflozin, on the inflammatory process is mediated via affection of TLR4/NLRP3 inflammasome signaling with subsequent repression of the inflammatory storm. Recently, the emerging role of SGLT2 inhibitors as potent anti-inflammatory and antioxidant agents in various neurological illnesses was attributed, at least partly, to their effects on RAGE signaling [42].

Hesperidin in the current work exerted potent modulatory effects on TLR4/RAGE/HMGB1 signaling which were evidenced by a significant decrease in the levels of NLRP3 inflammasome with significant attenuation of the inflammatory process in the brain. Muhammad et al. [43] stated that hesperidin and its derivatives ameliorate LPS-induced neuroinflammation and memory impairment by affecting TLR4/NF-κB signaling. In addition, Xie et al. [44] attributed the combatting effects of hesperidin on neuroinflammation to its modulatory influences on HMGB1/RAGE/NF-κB signaling with attenuation of the expression of NLRP3 inflammasome in the brain.

The results of the current work were in the same line with recent studies, which revealed that affection of PI3K/Akt/mTOR signaling is a key component of the pathogenic events that predispose to AD [45]. During the progression of AD, activated PI3K induces Akt phosphorylation with subsequent activation of mTOR which influences Tau phosphorylation and the amyloid cascade in the brain tissues. Additionally, activation of the PI3K/Akt/mTOR signaling pathway in AD leads to modulation of the autophagic pathways in the neuronal cells, thus aggravating the neurodegeneration [46]. Moreover, activation of PI3K/Akt signaling in AD enhances the nuclear translocation of NF-κB, which consequently increases the expression of the genes encoding the inflammatory cytokines leading to massive neuroinflammation [47].

In the present study, dapagliflozin significantly abrogated the effects of LPS on PI3K/Akt/mTOR signaling in the brain which coincided with Lin et al. [33] who postulated that the combatting effects of SGLT2 inhibitors against neurological disorders are derived from their ability to modulate PI3K/Akt/endothelial nitric oxide synthase signaling with subsequent affection of mTOR activation. These events were reflected in decreased production of ROS, downregulation of TLR4/RAGE/NF-κB signaling, and restoration of autophagy to normal levels. In addition, the ability of hesperidin to modulate PI3K/Akt/mTOR signaling with subsequent amelioration of neuroinflammation which was noticed in the present study was in the same line with Evans et al. [48] who highlighted the regulatory role of hesperidin and its derivatives on these signaling pathways which contributes efficiently to augmentation of autophagy and mitigation of the pathologic events induced by LPS.

The role of autophagy/apoptosis balance in the pathologic events of AD was thoroughly studied in the current research work [14]. Liu and Li [49] reported that cases with AD have significantly declined levels of the autophagy markers relative to the normal individuals which coincided with the findings of the present study. This defective autophagy was associated in the current work with significant upregulation of the apoptotic proteins and downregulation of the antiapoptotic molecules when compared to the control animals. This was explained by Condello et al. [50], who stated that apoptosis induction is usually coupled to autophagy inactivation. Caspase-3, which is a well-documented apoptotic marker, was found to cleave beclin-1, and hence destroy its proautophagic activity and exacerbate neurodegeneration [51]. Interestingly, the C-terminal fragment of beclin-1 resulting from this cleavage augments mitochondrial-mediated apoptosis. In addition, activation of caspase-3 increases the genetic expression of Atg4D, an enzyme that disrupts the autophagic activity of LC3-II [52].

In the present study, dapagliflozin induced significant disruption of apoptosis with significant enhancement of autophagy relative to the animals that received LPS alone. This was explained by the ability of dapagliflozin to combat impaired autophagy via affection of mTOR/NF-κB signaling [53]. Also, dapagliflozin was reported to inhibit apoptosis via the affection of the ERK1/2/cPLA2/AA/ROS pathway [54]. Another interesting finding in the current study was the effect of hesperidin on the restoration of the autophagic influx and suppression of apoptosis in the brain created by LPS. These effects were attributed to the potent antiapoptotic effects of hesperidin which were clearly obvious in neurodegenerative disorders such as AD [55]. Also, hesperidin, which is the aglycone part of hesperidin, was reported to modulate A1-42-induced autophagy via mechanisms related to PI3K/Akt and p38 mitogen-activated protein kinase signaling [48].

Ki-67 is a nuclear antigen that was reported to have an intimate relationship to the pathologic events of AD [16]. This was evidenced in the current work where the enhanced expression of Ki-67 in the brain induced by LPS was significantly combatted with the administration of each of dapagliflozin or hesperidin which signifies their role as cell cycle modulators in amelioration of the pathogenic events of AD.

The most favorable mitigating effects against LPS-induced AD were noticed in the present study with a dapagliflozin/hesperidin combination when compared against other groups that received either dapagliflozin or hesperidin alone. This might originate from the synergistic combatting effects possessed by both agents against the harmful consequences of oxidative stress created by LPS together with their ability to modulate the mediators of the inflammatory storm and restore the normal balance between autophagy and apoptosis. Additionally, the synergistic abrogating effects of both agents on HMGB1/TLR-4/RAGE signaling in the hippocampus were proven to affect the different pathogenic events that predispose to AD. Moreover, hesperidin exhibited inhibitory effects on p-glycoprotein in the different animal models [55,56]. This may explain the beneficial effects of the dapagliflozin/hesperidin combination, as dapagliflozin was reported to be a substrate for p-glycoprotein [57,58]. In view of the aforementioned findings, inhibition of p-glycoprotein expression by hesperidin with a subsequent increase in dapagliflozin bioavailability might explain the results encountered with a dapagliflozin/hesperidin combination.

## 4. Materials and Methods

### 4.1. Chemicals and Drugs

Santa Cruz Biotechnology, Inc., Dallas, TX, USA provided us with lipopolysaccharide, *Escherichia coli*, serotype O55:B5 (CAS number 93572-42-0). Dapagliflozin was purchased from SimSon Pharma Limited, Mumbai, India (CAS number 461432-26-8). Hesperidin was supplied by Cayman Chemical Co., Ann Arbor, MI, USA (CAS number 520-26-3). Dimethyl sulfoxide (DMSO) was purchased from Sigma-Aldrich Chemical Co., St Louis, MO, USA (CAS number 67-68-5). Normal saline was utilized for the dissolution of LPS. Both dapagliflozin and hesperidin were dissolved in a 10% DMSO solution. The other reagents used in the current study were supplied by SimSon Pharma Limited, Mumbai, India, and were of analytical grade.

### 4.2. The Experimental Design

Sixty male Wistar rats weighing about 150–230 g were utilized in the present study. Animals were housed in special cages with a temperature of 25 ± 4 °C, relative humidity of 54 ± 10%, and free access to food and water ad libitum with 12 h light/dark cycles. Animals were kept in these cages for ten days prior to any experiments to allow acclimatization. The U.K. Animals (Scientific Procedures Act, 1986), EU Directive 2010/63/EU for animal experiments were considered as a guide for designing the experiments and handling the animals. The Research Ethics Committee of the Faculty of Medicine, Tanta University, Egypt had approved the experimental protocol of the present study (approval code 36027/11/22). An individual who was blinded to the received treatments divided the animals randomly into six equal groups of ten rats each as follows: the control group was injected daily intraperitoneally with 0.5 mL 0.9% sodium chloride solution for one week; LPS group: LPS was injected daily intraperitoneally in a dose of 250 μg/kg for one week [59]; LPS + DMSO group: 1 mL of 10% DMSO was administered daily by oral gavage to LPS-injected rats; LPS + dapagliflozin group in which a daily dose of dapagliflozin (1 mg/kg) was administered by oral gavage to LPS-injected rats [60]; LPS + hesperidin group in which 80 mg hesperidin was administered daily by oral gavage to LPS-injected rats [61]; and LPS + dapagliflozin + hesperidin group in which the LPS-injected rats received dapagliflozin concomitantly with hesperidin daily orally in the aforementioned doses. Dapagliflozin, hesperidin, and DMSO administration started seven days prior to the beginning of and continued concomitantly with LPS injection until the end of the experimental period.

### 4.3. Determination of the Effect of Different Treatments on the Behavioral Tests

To determine the extent of memory and cognitive dysfunction in the animals exposed to the different treatments in the present study, the following behavioral tests were carried out starting from the 10th day after the beginning of the LPS injection.

#### 4.3.1. Open Field Locomotion Test (OFT)

In this test, rats were placed in a square black plexiglass apparatus in a room with dim light, and a video tracking system was used to record their movements for 5 min. Then, a video tracking analysis program for rodents obtained from Noldus, Wageningen, the Netherlands, was used for analysis of these recordings. Also, these recordings were utilized for manual calculation and scoring of the number of the rearing movements [62].

#### 4.3.2. Object Recognition Task

In this test, two different wooden block toys (A1 and A2) were put in a square black-painted wooden box with constant illumination and utilized as familiar objects to be discriminated by the animals. This test starts from the 10th day after the beginning of LPS injection and comprises three different stages, namely habituation, training, and testing stages. In the stage of habituation, adaption to the object recognition box was allowed to rats for 10 min. Later on, rats were placed inside the box with the two familiar objects 24 h after the habituation to give them an opportunity to explore these objects for 8 min. In the stage of testing, rats were placed in the testing box with one novel object and another familiar object for 8 min, and the sniffing time for both objects was recorded. Calculation of the discrimination index was performed by subtracting the time spent on the familiar object from the time spent on the novel one and the result was divided by the sum of time spent on the novel object and the time spent on the familiar one [63].

#### 4.3.3. Morris Water Maze Test

This test utilized a circular water-filled pool of 50 cm depth which has an escape platform submerged one centimeter below the water surface. Each animal had 3 training trials to find the platform per day starting from the 10th after the beginning of the LPS injection for five consecutive days. On the 6th day, the platform was removed. The time spent in the target quadrant for each animal was calculated by recording the swimming speed and the path length via a video tracking system obtained from Noldus, Wageningen, The Netherlands [64].

After performing the behavioral tests, isoflurane was used to anesthetize the animals. The skull was opened, and the brain was excised. Parts of the brain tissues were processed and subjected to histopathological and electron microscopic examination. Teflon homogenizer was utilized for homogenization of the hippocampal specimens which were then centrifuged by a cooling centrifuge at 1008× *g* for 20 min. Then, the biochemical parameters were assessed using the resulting supernatant.

### 4.4. Assessment of the Indicators of Oxidative Stress and Nuclear Factor Erythroid 2-Related Factor 2 (Nrf2) Content of the Hippocampal Tissue Specimens

Kits supplied by Wuhan Fine Biotech Co., Wuhan, Hubei, China (Catalog number ER1878) were utilized for the assessment of malondialdehyde (MDA) levels in the hippocampal tissues. The level of total antioxidant capacity of the hippocampal tissues was determined using kits obtained from Sunlong Biotech Co., Hangzhou, Zhejiang, China (Catalog number SL0776Ra). The levels of catalase (CAT) and superoxide dismutase (SOD) were measured in the hippocampal tissues using kits purchased from Shanghai Korain Biotech Co., Shanghai, China (code number E0869Ra and E1444Ra, respectively). In addition, paraoxonase-1 (PON1) levels were quantified in the hippocampal tissues using kits provided by ZellBio GmbH, Lonsee, Germany (Catalog number RK03898). Kits supplied by Northwest Life Science Specialities, LLC, Vancouver, WA, USA (Catalog number NFE2L2) were utilized for the measurement of Nrf2 content in the hippocampal tissues. Quantification of the aforementioned measurements was performed according to the vendor’s guide.

### 4.5. Assessment of Interleukin 1 Beta (IL-1β), IL-8, IL-18, and Monocyte Chemoattractant Protein-1 (MCP-1) in the Hippocampal Tissues

Kits purchased from Wuhan Fine Biotech Co., Wuhan, Hubei, China, were utilized for the measurement of tissue IL-1β and MCP-1 (catalog number ER1094 and ER0005, respectively). The hippocampal tissue content of IL-8 was quantified using kits supplied by Kamiya Biomedical Company, Seattle, WA, USA (catalog number KT-60204). Kits provided by Boster Biological Technology, Pleasanton, CA, USA (catalog number EK0592) were used for the determination of IL-18 content of the hippocampal tissues. The providers’ instructions were followed for the assessment of these parameters.

### 4.6. Quantification of Transforming Growth Factor Beta 1 (TGF-β1), TLR4, Nuclear Factor Kappa B (NF-κB), and NLRP3 Inflammasome Levels in the Hippocampal Tissues

Kits obtained from Diaclone SAS, Besançon Cedex, France (catalog number 670.020.192) were used for the determination of TGF-β1 content of the hippocampal tissues. Values of TLR4 in the hippocampal tissues were measured using kits supplied by Ozyme, Saint-Cyr-l’École, France (Catalog number RTEB0438). Wuhan Fine Biotech Co., Wuhan, Hubei, China supplied ELISA kits that were used for quantification of NF-κB and NLRP3 inflammasome in the hippocampal tissue specimens (catalog number ER1186 and ER1965, respectively). The vendors’ guidelines were followed for the quantification of the aforementioned biochemical parameters.

### 4.7. Assessment of the Levels of HMGB1 and RAGE in the Hippocampal Tissues

LSBio, Seattle, WA, USA was the supplier of ELISA kits (Catalog number LS-F4039-1) which were utilized for the determination of HMGB1 in the hippocampal tissues. RayBiotech, Peachtree Corners, GA, USA supplied the ELISA kits that were used for the measurement of the levels of RAGE in the hippocampal tissues (catalog number ELR-RAGE-1). Both HMGB1 and RAGE levels were determined following the provider’s instructions.

### 4.8. Measurement of Phospho-Akt, Phosphotylinosital-3-Kinase (PI3K), and Phospho-Mammalian Target of Rapamycin (p-mTOR) Levels in the Hippocampal Tissues

Determination of PI3K levels in the hippocampal tissues was performed using ELISA kits purchased from Cosmo Bio USA, Carlsbad, CA, USA (catalog number CSB-E08418r-1). Detection of the phosphorylated form of Akt (pS473) and the total Akt levels was carried out in the current study using kits provided by RayBiotech, Peachtree Corners, GA, USA (catalog number PEL-Akt-S473-T-5). Likewise, kits purchased from Abcam, Waltham, MA, USA (catalog number ab279869) were used for assessment of the phosphorylated form of mTOR (Phospho-Ser2481) and the total mTOR levels in the hippocampal tissues. The providers’ guidelines were followed for the quantification of the aforementioned biochemical parameters.

### 4.9. Assay of the Levels of Beclin-1 and LC3-II in the Hippocampal Tissues

MyBioSource, San Diego, CA, USA supplied ELISA kits (catalog number MBS2706719) which were used for evaluation of the levels of beclin-1 in the hippocampal tissues. Antibodies-online GmbH, Aachen, Germany supplied ELISA kits (catalog number ABIN6938806) that were used for the assessment of LC3-II levels in the hippocampal tissues. Both autophagy markers were assessed according to the vendors’ instructions.

### 4.10. Quantification of Caspase 3, Caspase 9, and B-Cell Lymphoma (BCL-2) Protein Levels in the Hippocampal Tissues

In the current study, kits purchased from Shanghai Korain Biotech Co., Shanghai, China were utilized for quantitative assessment of tissue caspase 3 (code number E1648Ra). Antibodies-online GmbH, Aachen, Germany supplied ELISA kits (catalog number ABIN771575) that were used for assessment of caspase 9 levels in the hippocampal tissues. ELISA kits obtained from Biorbyt Ltd., Cambridge, UK (catalog number orb567225) were used for the assessment of BCL-2 levels in the hippocampal tissues. The vendors’ instructions were obeyed to assess the aforementioned apoptosis markers.

### 4.11. Microscopic Detection of the Histopathological Changes of the Brain Tissues

After extraction of the brain, neutral buffered formalin (10%) was used for immediate fixation of the frontal lobe and the hippocampus. After that, these specimens were immersed in paraffin-forming blocks which were cut to yield 5 µm thickness sections and stained with hematoxylin and eosin (H&E). After completion of the staining process, these sections were examined using a light microscope (Olympus, Tokyo, Japan).

### 4.12. Determination of the Immunohistochemical Expression of Protein Expression of Ki-67 in the Brain Tissues

The slides containing the prepared brain tissue sections were dewaxed and then rehydrated for 10 min. After that, the endogenous peroxidase activity was blocked using 3% hydrogen peroxide. The Ki-67 epitope was recovered by using the heat-induced epitope retrieval method in citrate buffer (pH = 6) with incubation for three minutes in a microwave oven. Then, the slides were incubated for two hours with primary antibodies Ki-67-clone SP6 obtained from Abcam, Waltham, MA, USA (Catalog number ab16667) followed by application of biotinized secondary antibodies (Biorbyt Ltd., Cambridge, UK, catalog number orb389335). The immunoreactivity for Ki-67 was visualized by utilizing extravidin peroxidase and 3,3′-diaminobenzidine as chromogen purchased from Sigma-Aldrich Co., St. Louis, MO, USA. The immunoexpression of Ki-67 was detected under a light microscope (Olympus, Japan) and was graded as follows: (+) denotes mild immunoexpression of Ki-67; (++) denotes moderate immunoexpression of Ki-67; and (+++) refers to massive immunoexpression of Ki-67 in the brain tissues [65].

### 4.13. Examination of the Electron Microscopic Morphologic Changes in the Brain Tissues

Glutaraldehyde 4% in 0.1 M cacodylate buffer (pH 7.4) was utilized for fixation of the specimens extracted from the frontal lobe for 48 hrs at 4 °C. Thereafter, these specimens were sectioned into small parts and washed with distilled water. Then, fixation of these small portions in 1% osmium tetraoxide with 15 mg/mL of potassium ferrocyanide was performed for a couple of hours at 4 °C. An ultramicrotome was used to cut these fixed specimens into 1 μm thickness sections. After cutting, these tiny sections were stained with uranyl acetate and lead, and the transmission electron microscope (Hitachi-7650; Hitachi, Tokyo, Japan) was used to detect the electron microscopic morphologic changes that took place in the brain tissues extracted from the different groups.

### 4.14. Statistical Comparisons between the Different Groups

Data presented in this study were statistically analyzed using GraphPad Prism, version 7 (GraphPad Software, LLC, La Jolla, CA, USA). The different groups were compared to each other using a one-way analysis of variance (one-way ANOVA) followed by Dunnett’s post hoc test which was utilized as a confirmatory test. These data were presented as mean ± standard deviation (SD). The significance level was set at a level of *p*-value less than 0.05.

## 5. Conclusions

**Figure 16:**
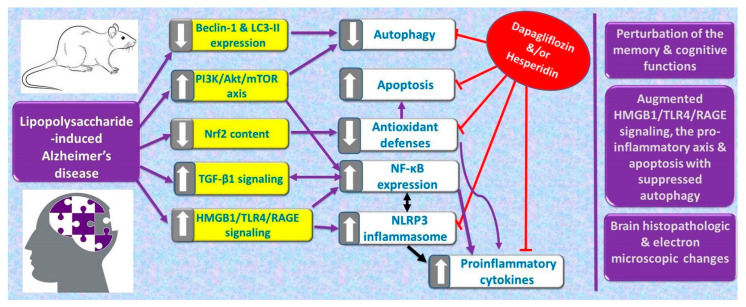
Summary of the possible mechanisms by which dapagliflozin and/or hesperidin might combat the pathologic changes in the brain tissues elicited by lipopolysaccharide injection.

Inhibition of the hippocampal HMGB1/TLR-4/RAGE signaling, the pro-inflammatory axis, and apoptosis alongside augmentation of the antioxidant defenses and autophagy can be regarded as beneficial effects by which dapagliflozin/hesperidin combination may combat LPS-triggered AD (Figure 16). Further research is vitally needed to investigate in-depth the consequences of targeting the aforementioned pathways by dapagliflozin/hesperidin combination and to evaluate the clinical significance of the application of these results.

## Data Availability

Data are available from the corresponding author upon reasonable request.
